# Fetal biometry assessment with Intergrowth 21st’s and Salomon’s equations in rural Burkina Faso

**DOI:** 10.1186/s12884-020-03183-5

**Published:** 2020-08-26

**Authors:** Biébo Bihoun, Serge Henri Zango, Maminata Traoré-Coulibaly, Innocent Valea, Raffaella Ravinetto, Jean-Pierre Van Geertruyden, Umberto D’Alessandro, Halidou Tinto, Annie Robert

**Affiliations:** 1 IRSS-Clinical Research Unit of Nanoro, Nanoro, Burkina Faso; 2 Intitut de recherche expérimentale et clinique, Université catholique de Louvain, Brussels, Belgium; 3 Public Health Department, Institute of Tropical Medicine, Antwerp, Belgium; 4 Global Health Institute, University of Antwerp, Antwerp, Belgium; 5 Medical Research Council Unit The Gambia at the London School of Hygiene and Tropical Medicine, London, UK

**Keywords:** Biometry, References, Standards, Burkina Faso

## Abstract

**Background:**

Ultrasound scanning during the 2nd or the 3rd trimester of pregnancy for fetal size disturbances screening is heavily dependent of the choice of the reference chart. This study aimed to assess the agreement of Salomon and the Intergrowth 21st equations in evaluating fetal biometric measurements in a rural area of Burkina Faso, and to measure the effect of changing a reference chart.

**Methods:**

Data collected in Nazoanga, Burkina Faso, between October 2010 and October 2012, during a clinical trial evaluating the safety and efficacy of several antimalarial treatments in pregnant women were analyzed. We included singleton pregnancies at 16–36 weeks gestation as determined by ultrasound measurements of fetal bi-parietal diameter (BPD), head circumference (HC), abdominal circumference (AC) and femur length (FL). Expected mean and standard deviation at a given gestational age was computed using equations from Salomon references and using Intergrowth 21st standard. Then, z-scores were calculated and used subsequently to compare Salomon references with Intergrowth 21st standards.

**Results:**

The analysis included 276 singleton pregnancies.

Agreement was poor except for HC: mean difference − 0.01, limits of agreement − 0.60 and 0.59. When AC was used as a surrogate of fetal size, switching from the reference of Salomon to the standards of Intergrowth 21st increased ten times the proportion of fetuses above the 90th percentile: 2.9 and 31.2%, respectively.

Mean differences were larger in the third trimester than in the second trimester. However, agreement remained good for HC in both trimesters.

Difference in the proportion of AC measurements above the 90th percentile using Salomon and Intergrowth 21st equations was greater in the second trimester (2.6 and 36.3%, respectively) than in the third trimester (3.5 and 19.8%, respectively). The greatest difference between the two charts was observed in the number of FL measurements classified as large in the second trimester (6.8 and 54.2%, using Salomon and Intergrowth 21st equations, respectively).

**Conclusion:**

The agreement between Intergrowth 21st and Salomon equations is poor apart from HC. This would imply different clinical decision regarding the management of the pregnancy.

## Background

Ultrasound scanning during the 2nd or 3rd trimester of pregnancy allows fetal anthropometrics measurement [1] and screening for fetal size disturbances by comparison to reference values [2, 3].

Biparietal diameter (BPD), head circumference (HC), abdominal circumference (AC), and femur length (FL) are the most commonly measured parameters [4, 5].

Abnormal fetal biometric measurements could reflect underlying health issues like microcephalia, aneuploidy, genetic syndrome of skeletal dysplasia [6].

Early detection of abnormal fetal size helps obstetrician initiating further monitoring, planning and managing delivery in terms of labor induction or caesarian section [6, 7].

However, abnormal fetal size identification depends heavily on the choice of reference values. There is more than eighty references charts in the world [4]. A switching from one reference chart to another could raise tenfold the likeliness of identifying abnormal biometric measurements [8]. This could lead to expensive and stressful monitoring with additional investigations [8, 9]. Also, fetal size depends of ethnicity and use of inadequate reference may result in harmful medical decisions [10, 11]. For example, there is a risk of fetal loss in cases in which karyotype is demanded because of the invasive sampling method used [8]. Screening for fetal size disturbances using inappropriate reference chart may affect research conclusions and health policies as well [8, 10]. Thus some authors recommended to use local reference for screening in a specific population [11–14]. In high-income countries, [4, 5] local biometric reference charts were adopted. Clear guidelines and recommendations for screening of abnormal fetal size and subsequent management were also put in place [5, 15–21]. In low- and middle-income countries (LMICs), and particularly in sub-Saharan Africa, local charts are either lacking or not implemented where available [22–25]. The latter mostly refer to European charts [26], or charts preprogramed by default in the ultrasound device software [11–14, 27].

In 2014, the international fetal and newborn growth consortium for the twenty-first century (Intergrowth 21st) published fetal biometric standard equations based on selected healthy pregnancies from eight countries [10, 27]. The aim was to provide charts that could be used anywhere in the world and to solve the issue of inadequate references [27]. Settings where local reference charts are lacking or not implemented are likely to replace the charts they are currently using by this new chart. However, knowing the variation between nomograms in assessing fetal size and the clinical implication, it would be cautious to check whether adaptation is needed before adopting or replacing a chart in a specific population [14].

In Burkina Faso, no locally-adapted fetal biometry charts have been adopted or recommended in obstetric ultrasound practice. Rather, French references or other European references set by default in the ultrasound machines are used. Indeed in France, Salomon equations were recommended until 2018 [28].

Both the Salomon and Intergrowth 21st equations allow the calculation of a mean and a standard deviation by gestational age and the computation of a standardized z-score for the individual fetus. The objective of the present analysis was to assess the difference between z-scores derived from Salomon and from Intergrowth 21st equations using fetal biometric measurements in pregnant women from rural areas of Burkina Faso, and to measure the effect of changing a reference chart.

## Methods

### Study settings and population

The current dataset is from the trial “Safe and efficacious artemisinin-based combination treatment for African pregnant women with malaria” (PREGACT) conducted from June 2010 to August 2013 [NCT00852423 (ClinicalTrials.gov)]. The primary study evaluated the efficacy and safety of four artemisinin-based combinations treatment in women with malaria in the 2nd and 3rd trimester of pregnancy. The trial was implemented in four countries, namely Burkina Faso, Ghana, Malawi, and Zambia. Methods and results have been already published [29, 30]. This analysis used data collected in Nazoanga, Burkina Faso, where fetal biometric measurements at inclusion by ultrasound were carried out, to exclude women in the 1st trimester.

### Ultrasound

This study was cross-sectional. Ultrasonographic examination of the pregnant women was performed once at inclusion. However, three participants had their scan repeated a second time at screening to confirm gestational age as per the quality assurance system put in place [31, 32]. A Fukuda Denshi© portable ultrasound scanner FFsonic UF-4100 with a 3.5 MHz or 5.0 MHz probes was used for transabdominal examination according to woman’s thinness.

Four biometric parameters were measured. BPD and HC were both obtained in a transverse view of the fetal head with the following landmarks: midline echo corresponding to the fax cerebri, its anterior third interrupted by the cavum septi pellucidi, symmetry of thalami at each side. BPD was measured from the inner to the outer wall of the skull. HC measurement was realized by placing an ellipse around the outer border of the skull. AC was measured by applying the ellipse on the external border of the abdomen in a cross-sectional plane showing the stomach bulbe and the anterior third of the umbilical vein. FL measurement was obtained in a plane where the femoral diaphysis was fully visible, with calipers placed on the both ends. All measurements were done according to specific standard operating procedures [31, 32]. Gestational age in complete weeks was automatically derived from the four anthropometric measurements according to Hadlock formula [32, 33]

### Statistical analysis

Three participants have their scan repeated once at screening to confirm gestational age as per the quality assurance system [29, 30]. We took this into account by averaging the two measurements for each biometric parameter.

The expected mean and expected standard deviation for BPD, HC, AC and FL for the gestational age were computed for each fetus using equations from Salomon [16] and Intergrowth 21st [27]. The z-scores [(Observed value – Expected mean)/Expected SD] of the two equations were compared. Square-diagonal scatter plots were drawn to allow visual evaluation of the relationship between the two sets of z-scores. All differences (Intergrowth 21st z-score - Salomon z-score) were compared using paired t test and Wilcoxon signed ranks test. A *p*-value < 0.05 was considered as statistically significant. Linear relationship was checked by performing linear regression of Intergrowth 21st z-scores by Salomon z-scores.

Level of agreement between the charts was checked using Bland-Altman analysis. Individual scores averages were plotted horizontally against their differences vertically. Limits of agreement (LOA) were obtained by applying the following formula: mean of differences ±1.96 standard deviation.

A mean difference of zero and limits of agreement within − 0.50 and 0.50 were considered as a good agreement [34].

Reliability between the two charts was expressed by the intraclass correlation coefficient (ICC), calculated from a random effect one-way analysis of variance. Reliability was considered as weak, good or excellent if ICC values were <  0.40, between 0.40 and 0.75, or > 0.75, respectively.

Abnormal measurement referred to either smallness (z score < − 2.00 corresponding to the 2.5th centile, z score < − 1.282 corresponding to the 10th centile) or largeness (z-score > 1.282 corresponding to the 90th centile, or z-score > 2.00 corresponding to the 97.5th centile).

The effect of replacing one chart with another was measured using AC as a surrogate of fetal weight [2, 9].

STATA® statistical software version 15.1 StataCorp LLC, Texas, USA, was used for all analyses.

### Ethics

Ethical approvals were obtained for the PREGACT study from the ethics committee of the University of Antwerp, Belgium, the institutional ethics committee of the Centre Muraz and the ethics committee of the Ministry of Health, Burkina Faso. Study participants or their legally authorized representative (for minors/not emancipated) signed (or thumb printed if illiterate) an informed consent form, before entering the study [29, 30]. The data were anonymized.

## Results

Out of the 285 pregnant women recruited in Nazoanga, 9 were excluded: 6 because of consent withdrawal and 3 because of twin pregnancy. Therefore, 276 participants were included in the current analysis. Recruited women were young (median age: 23 years), had several pregnancies (median gravidity: 3), and at inclusion had a median gestational age of 25 weeks (Table 1).

**Table 1:** General characteristics of the mothers

Age (years)	23 (20; 29)
Gravidity	3 (1; 4)
Parity	2 (0; 3)
Weight (kg)	54 ± 6
Height (cm)	162 ± 7
Body mass index (kg/m^2^)	20.5 ± 1.7
Symphysis-fundal height (cm)	24 (21; 28)
Gestational age (weeks)	25 (21; 29)
Hemoglobin (g/dL)	10.1 ± 1.2

Numbers are mean ± standard deviation or median (IQR)

*IQR* interquartile range

**Fig. 1:**
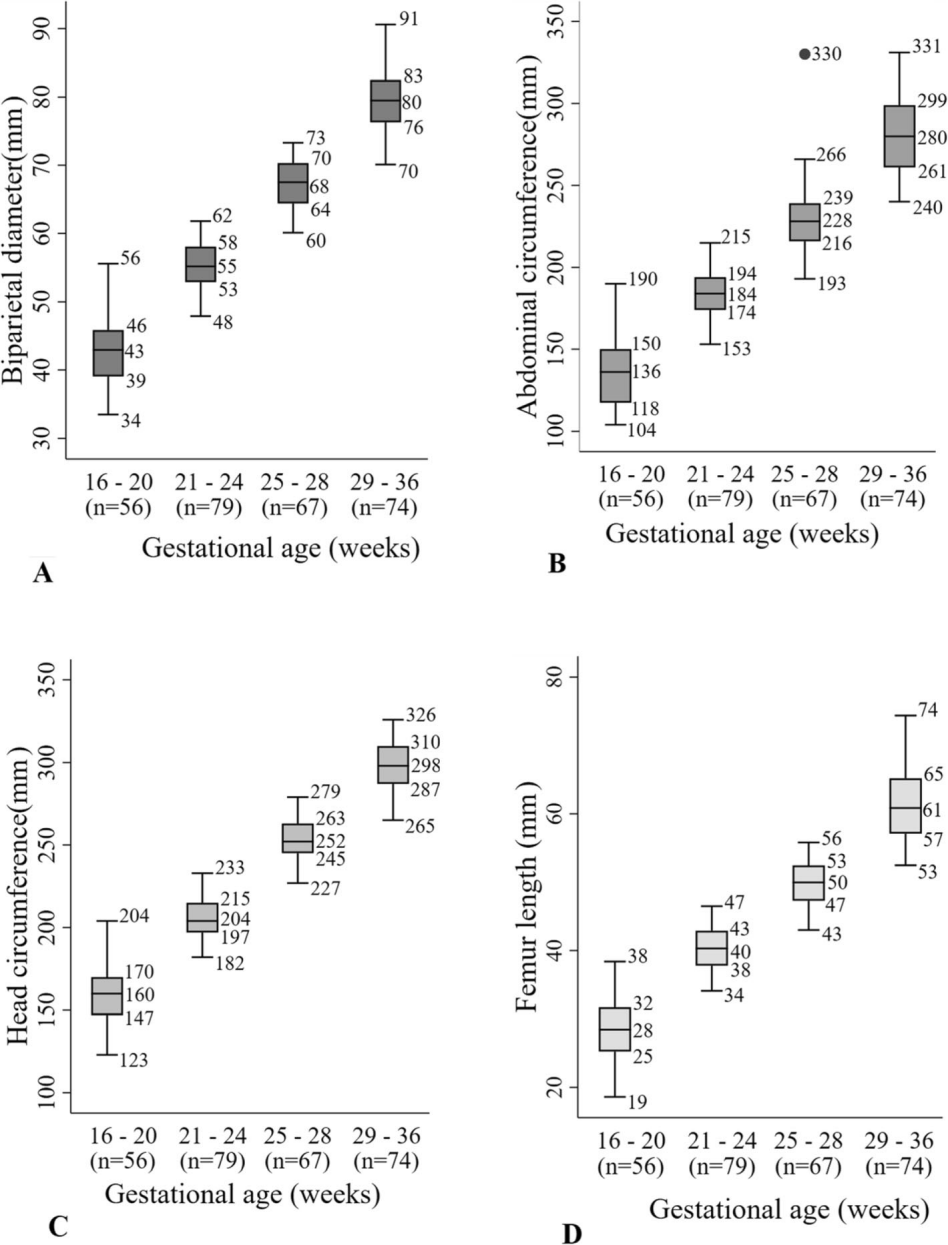
Fetal biometry measurements by gestational age. **a**: Biparietal diameter. **b**: Abdominal circumference. **c**: Head circumference. **d**: Femur length. Intergrowth 21: International fetal and newborn growth consortium for the twenty-first century. The numbers at the right of the boxplots represented from the bottom to the top: the minimum without outliers, the first quartile, the median, the third quartile and the maximum without outliers

Median and interquartile range (IQR) of the measured parameters increased with increasing gestational age (Fig. 1).

**Fig. 2:**
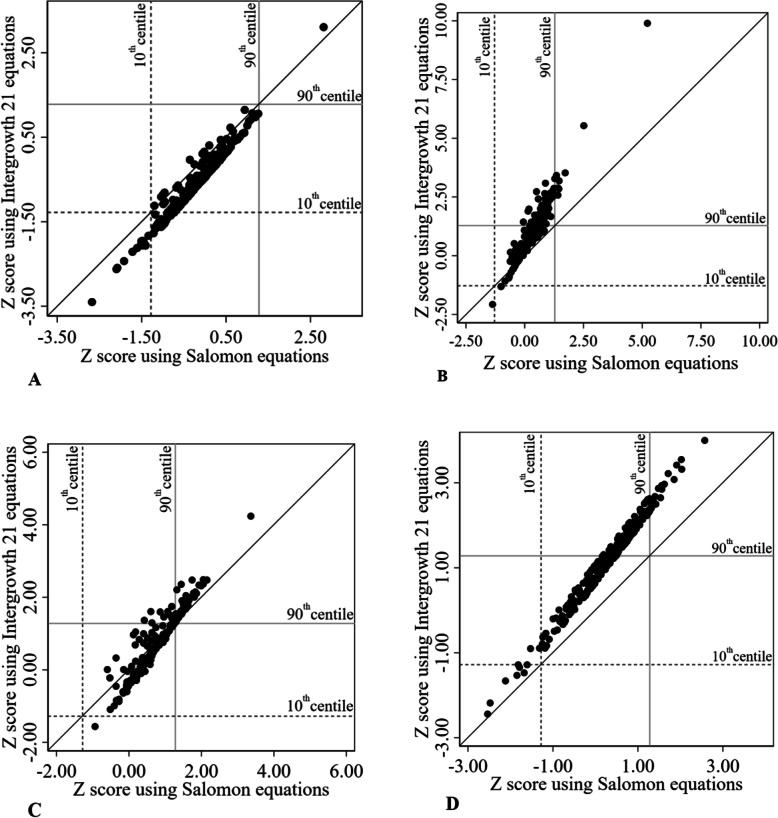
Number of standard deviations from the mean using Salomon or Intergrowth 21 eqs. **a**: Biparietal diameter. **b**: Abdominal circumference. **c**: Head circumference. **d**: Femur length. Intergrowth 21: International fetal and newborn growth consortium for the twenty-first century. Dashed horizontal and vertical grey lines referred to a z score of − 1.282 corresponding to the 10th centile. Solid horizontal and vertical grey lines referred to a z score of 1.282 corresponding to the 90th centile. The black oblique line is the perfect concordance line where the z-scores from Intergrowth 21st and Salomon equations are equal

Visual comparison by scatter plots of Intergrowth 21st ‘s to Salomon’s z scores showed that they were underestimated in the low values of BPD (Fig. 2a), AC (Fig. 2b), and HC (Fig. 2c), and overestimated in the high values of AC (Fig. 2b), and HC (Fig. 2c); all z scores of FL were overestimated (Fig. 2d).

**Fig. 3:**
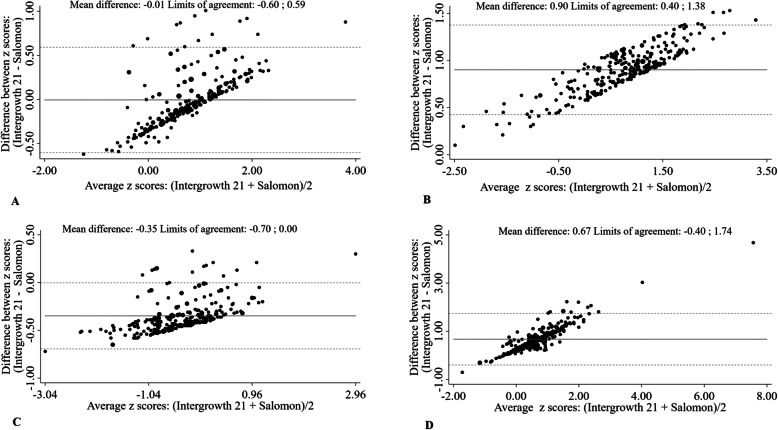
Agreement between z scores using Salomon or Intergrowth 21 equations. **a**: Head circumference. **b**: Femur length. **c**: Biparietal diameter. **d**: Abdominal circumference. Intergrowth 21: International fetal and newborn growth consortium for the twenty-first century. Dashed horizontal grey lower line represents the lower limit of agreement between the z-scores from Intergrowth 21st and Salomon equations. Solid horizontal grey line represents the mean difference between the z-scores from Intergrowth 21st and Salomon equations. Dashed horizontal grey upper line represents the upper limit of agreement between the z-scores from Intergrowth 21st and Salomon equations

The two sets of z scores agreed poorly except for HC. The mean difference (− 0.01) was closed to zero and the limits of agreement (− 0.60 and 0.59) were closed to the prespecified values of − 0.5 and 0.5. (Fig. 3a). Reliability ranged from good to excellent (see Additional file 1, Supplemental Table 1).

**Fig. 4:**
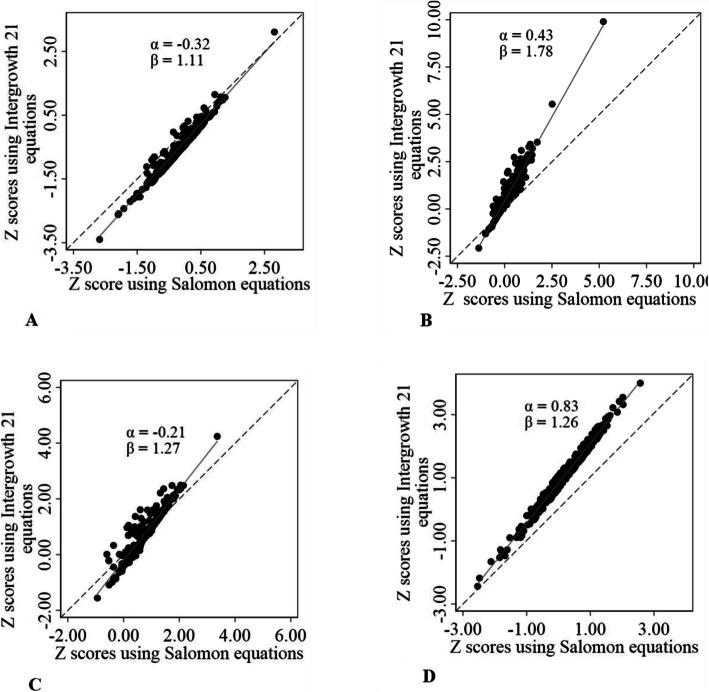
Regression of Intergrowth 21 z-scores with Salomon z-scores. **a**: Biparietal diameter. **b**: Abdominal circumference. **c**: Head circumference. **d**: Femur length. Intergrowth 21: International fetal and newborn growth consortium for the twenty-first century. Dashed oblique black line represents the perfect agreement between the z-scores from Intergrowth 21st and Salomon equations. Solid oblique grey line represents the linear regression fitted line

There was a strong linear correlation between the z scores by Intergrowth 21st equations and the z scores by Salomon equations. The slopes of linear regression of z scores using Intergrowth 21st equations over the z scores using Salomon equations ranged from 1.11 for BPD to 1.78 for AC (Fig. 4).

The percentages of fetal anthropometrics classified either as small or large are reported in Table 2. Globally, the number of measurements considered as large was greater than that of measurements considered as small, except for BPD. Also, percentages of fetuses with abnormal z scores by Intergrowth 21st equations were higher than those by Salomon equations. The effect of replacing Salomon reference by Intergrowth 21st standards was shown using AC as surrogate of fetal size. Large fetuses (above the 90th percentile) proportion using Salomon eqs. (2.9%) was decupled when Intergrowth 21st equations were used (31.2%).

**Table 2:** Fetuses with abnormal z scores using Intergrowth 21st or Salomon equations

Anthropometrics	Abnormal z score n (%)			
	< 2.5th centile	< 10th centile	> 90th centile	> 97.5th centile
Biparietal diameter				
Intergrowth 21	10 (3.6)	39 (14.1)	1 (0.4)	1 (0.4)
Salomon	3 (1.1)	14 (5.1)	1 (0.4)	1 (0.4)
Mc Nemar test	n.a	< 0.001	n.a	n.a
Abdominal circumference				
Intergrowth 21	1 (0.4)	3 (1.1)	86 (31.2)	33 (12.0)
Salomon	0 (0.0)	1 (0.4)	8 (2.9)	2 (0.7)
Mc Nemar test	n.a	n.a	< 0.001	< 0.001
Head circumference				
Intergrowth 21	0 (0.0)	1 (0.4)	68 (24.6)	19 (6.9)
Salomon	0 (0.0)	0 (0.0)	52 (18.8)	4 (1.4)
Mc Nemar test	n.a	n.a	< 0.001	< 0.001
Femur length				
Intergrowth 21	2 (0.7)	6 (2.2)	137 (49.6)	49 (17.8)
Salomon	3 (1.1)	10 (3.6)	18 (6.5)	3 (1.1)
Mc Nemar test	n.a	n.a	< 0.001	< 0.001

*n.a* not applicable; Intergrowth 21: international fetal and newborn growth consortium for the twenty-first century

In the second trimester, the agreement between HC z scores using Intergrowth 21st equations and z scores using Salomon equations remained good: mean difference 0.03; limits of agreement − 0.62 and 0.68 (Table 3). The proportions of large fetuses based on AC measurements above the 90th percentile were 36.3% by Intergrowth 21st equations and 2.6% by Salomon equations. However, the greatest difference in large biometric measurements between the four parameters was observed in FL (6.8% and 54. 2%, using Salomon and Intergrowth 21st equations respectively) (Table 4).

**Table 3:** Agreement and reliability of fetal biometrics z scores using Intergrowth 21st and Salomon equations in the second trimester

Anthropometrics	Mean ± SD	p-value^*^	Median (IQR)	p-value^†^	Range
Biparietal diameter					
Intergrowth 21	− 0.48 ± 0.72		− 0.47 (− 0.98; − 0.04)		−3.40; 3.11
Salomon	− 0.15 ± 0.63		− 0.12 (− 0.60; 0.24)		−2.68; 2.81
Difference	− 0.33 ± 0.21	< 0.001	− 0.40 (− 0.48; − 0.22)	< 0.001	− 0.72; 0.33
LOA	− 0.74; 0.07				
ICC	0.85				
Abdominal circumference					
Intergrowth 21	1.05 ± 1.18		0.95 (0.35; 1.59)		−2.07; 9.90
Salomon	0.29 ± 0.63		0.23 (−0.03; 0.58)		−1.37; 5.22
Difference	0.77 ± 0.60	< 0.001	0.69 (0.40; 1.01)	< 0.001	− 0.70; 4.68
LOA	− 0.40; 1.94				
ICC^‡^	0.54				
Head circumference					
Intergrowth 21	0.75 ± 0.81		0.72 (0.14; 1.29)		−1.56; 4.24
Salomon	0.72 ± 0.61		0.67 (0.31; 1.14)		−0.94; 3.36
Difference	0.03 ± 0.33	0.18	−0.02 (− 0.22; 0.23)	0.85	−0.62; 1.01
LOA	−0.62; 0.68				
ICC	0.89				
Femur length					
Intergrowth 21	1.21 ± 0.92		1.34 (0.63; 1.81)		−1.53; 4.00
Salomon	0.36 ± 0.71		0.47 (−0.07; 0.77)		−1.85; 2.57
Difference	0.85 ± 0.21	< 0.001	0.88 (0.73; 0.97)	< 0.001	0.21; 1.43
LOA	0.43; 1.27				
ICC	0.63				

* Paired t test *p* value

† Wilcoxon signed ranks test *p* value

^‡^ Intraclass correlation calculation for abdominal circumference z scores excluded one participant with extreme values (9.9 using Intergrowth equations and 5.22 using Salomon equations)

*LOA* limits of agreement, *ICC* intraclass correlation coefficient

**Table 4:** Abnormal z scores using Intergrowth 21st or Salomon equations in the second trimester

Anthropometrics	Abnormal z scores n (%)			
	< 2.5th centile	< 10th centile	> 90th centile	> 97.5th centile
Biparietal diameter				
Intergrowth 21	4 (2.1)	16 (8.4)	1 (0.5)	1 (0.5)
Salomon	1 (0.5)	4 (2.1)	1 (0.5)	1 (0.5)
Abdominal circumference				
Intergrowth 21	1 (0.5)	3 (1.6)	69 (36.3)	28 (14.7)
Salomon	0 (0)	1 (0.5)	5 (2.6)	2 (1.1)
Head circumference				
Intergrowth 21	0 (0)	1 (0.5)	49 (25.8)	15 (7.9)
Salomon	0 (0)	0 (0)	34 (17.9)	3 (1.6)
Femur length				
Intergrowth 21	0 (0)	2 (1.1)	103 (54.2)	32 (16.8)
Salomon	0 (0)	4 (2.1)	13 (6.8)	2 (1.1)

In the third trimester mean difference between HC z scores was − 0.09 and limits of agreement were − 0.52 and 0.35 (Table 5). Large fetuses detected by AC z scores above the 90th percentiles were 19.8 and 3.5% using Intergrowth 21st and Salomon equations, respectively (Table 6).

**Table 5:** Agreement and reliability of fetal biometrics z scores using Intergrowth 21st and Salomon equations in the third trimester

Anthropometrics	Mean ± SD	p-value^*^	Median (IQR)	p-value^†^	Range
Biparietal diameter					
Intergrowth 21	−0.91 ± 0.70		−0.89 (−1.31; −0.44)		−2.62; 0.78
Salomon	− 0.53 ± 0.64		−0.49 (− 0.90; − 0.11)		−2.10; 1.00
Difference	−0.39 ± 0.07	< 0.001	−0.38 (− 0.44; − 0.34)	< 0.001	−0.53; − 0.22
LOA	−0.52; − 0.25				
ICC	0.85				
Abdominal circumference					
Intergrowth 21	0.81 ± 0.71		0.75 (0.29; 1.21)		−0.95; 2.89
Salomon	0.35 ± 0.43		0.36 (0.02; 0.63)		−0.68; 1.45
Difference	0.45 ± 0.31	< 0.001	0.37 (0.25; 0.64)	< 0.001	−0.27; 1.48
LOA	−0.16; 1.07				
ICC	0.66				
Head circumference					
Intergrowth 21	0.76 ± 0.70		0.70 (0.39; 1.21)		−0.87; 2.49
Salomon	0.85 ± 0.48		0.79 (0.58; 1.17)		−0.28; 2.05
Difference	−0.09 ± 0.22	< 0.001	− 0.09 (− 0.18; 0.07)	0.001	−0.59; 0.44
LOA	−0.52; 0.35				
ICC	0.92				
Femur length					
Intergrowth 21	1.09 ± 1.15		1.15 (0.53; 1.75)		−2.44; 3.55
Salomon	0.07 ± 0.90		0.13 (−0.38; 0.55)		− 2.54; 2.02
Difference	1.02 ± 0.26	< 0.001	1.04 (0.91; 1.16)	< 0.001	0.10; 1.53
LOA	0.50; 1.53				
ICC	0.65				

* Paired t test *p* value

† Wilcoxon signed ranks test *p* value

*LOA* limits of agreement, *ICC* intraclass correlation coefficient

**Table 6:** Abnormal z scores using Intergrowth 21st or Salomon equations in the third trimester

Anthropometrics	Abnormal z scores n (%)			
	< 2.5th centile	< 10th centile	> 90th centile	> 97.5th centile
Biparietal diameter				
Intergrowth 21	6 (7.0)	23 (26.7)	0(0)	0(0)
Salomon	2 (2.3)	10 (11.6)	0(0)	0(0)
Abdominal circumference				
Intergrowth 21	0(0)	0 (0)	17 (19.8)	5 (5.8)
Salomon	0(0)	0 (0)	3 (3.5)	0(0)
Head circumference				
Intergrowth 21	0(0)	0 (0)	19 (22.1)	4 (4.7)
Salomon	0(0)	0 (0)	18 (20.9)	1 (1.2)
Femur length				
Intergrowth 21	2 (2.3)	4 (4.7)	34 (39.5)	17 (19.8)
Salomon	3 (3.5)	6 (7.0)	5 (5.8)	1 (1.2)

## Discussion

The aim of our study was to determine the differences between fetuses’ size patterns estimated by Salomon references or Intergrowth 21st standards in a sub-Saharan African rural population, rather than estimating the actual status of smallness or largeness.

The differences between the means of z-scores of the four biometric parameters estimated by the two methods were all statistically significant. Intergrowth 21st equations gave the greatest scores, particularly for FL. Therefore, the charts agreed poorly, except for HC.

These findings revealed differences between our population and the populations used for the charts [1, 10]. Indeed, Salomon‘s chart was developed on the basis of a cohort of pregnant women followed up in France which probably is ethnically different from our cohort of Burkinabe pregnant women, [16] possibly explaining the observed discrepancies [12–14]. Nevertheless, the development of Intergrowth 21st equations included African pregnant women [27] but had greater means of z-scores, meaning that other factors than ethnicity could explain the differences observed. Intergrowth 21st equations were derived on the basis of healthy, well-nourished women [10] and thus describe growth under optimal conditions [1, 10]. References of Salomon imposed few constraints regarding adequacy of the nutritional or health status [16]. Our study population included malaria-infected pregnant women living in rural Burkina Faso [29]; on average observed measures were more distant from standards and closer to references, suggesting that ideal fetal growth conditions were not fulfilled. Nevertheless, the negative means of BPD z-scores were probably due to systematic variations in head measurement methods as already shown in another publication [34]. BPD was obtained by placing calipers in the center of the width of the skull bone, from outer-to-outer and from outer-to-inner margins, in the study of Salomon’s, Intergrowth 21st study and in our study [16, 27, 32].

Despite these disparities, both charts agreed roughly on HC measurements. This finding reinforces the choice of HC as a single “non-fat marker” for comparison of fetal size across populations [4, 10].

It was recently shown that FL z scores between Intergrowth 21st and Salomon’s equations were largely divergent in France, [34] a difference also observed in our study that may be due to the evolution in ultrasound technology [35]. Indeed, recent ultrasound equipment’s such as those used in the Intergrowth 21st study have thinner beam and yield smaller FL [35, 36] than older machines as in our study [31] and in Salomon’s [16].

When AC was used as a proxy of fetal size estimation [2], the proportions of small fetuses were low for both charts which may indicate the difficulty ultrasonography has in identifying small fetuses [15, 37]. However, the proportions of both small and large fetuses were higher with Intergrowth 21st equations than with Salomon equations and a similar trend was remarked with HC, suggesting the tendency of Intergrowth 21st equations to underestimate the size in small measurements and to overestimate it in high measurements [3, 34]. Thus, the choice of one or another of these references would implies very different medical interventions, follow-up, and resources allocation as well as stress put on patients [4, 27].

It is well documented that pregnancies affected by malaria, as in our study, are subject to fetal growth restriction [31, 38]. Thus, the number of small fetuses was expected to be high even if differences would be found between the charts. Surprisingly, this number was very low and the number of large fetuses was high. We suspect gestational age determination to be a possible cause of such situation. Indeed, pregnancies were dated late owing to the study design, [29] using a combination of fetal biometry measurements [33]. Late dating is less accurate than early dating. However, this is common practice in sub-Saharan Africa where almost three out of four pregnant women attend their first antenatal clinic during the second or third trimester, or not at all [39]. Although the combination of fetal anthropometric measurement is the recommended method at this stage of pregnancy, [40] it could produce redundant relationship when used for determining both gestational age and fetal size [4]. This is of particular concern in areas where malaria in pregnancy is common such as in Burkina Faso [41]. Gestational age could be underestimated in case of symmetric fetal growth restriction [33], hiding the adverse effect of malaria [42]. However, the difficulty for estimating the gestational age applies to both sets of equations when calculating z scores. Therefore, the differences between the charts are probably not due only to pregnancy dating problems, as shown by the positive and significant slopes in linear regressions. In addition, pregnant women included in this clinical trial, besides malaria, did not have any other chronic or major disease with adverse effect on fetal growth [29].

This is a post-hoc analysis and thus, has some limitations. Physicians performing the ultrasound scans are not professional sonographers even if trained ad hoc. In addition, the study design was not conceived to evaluate two different methods for the assessment of fetal biometry. Moreover, the use of European references equations may not be appropriate for African populations. Gestational age determined in late pregnancy is also another limitation because of less accuracy. Our study population prone to malaria was quite selected and this maybe introduce a bias. However, a recent study showed that the difference between the two charts remained while using fetal biometric measurements from pregnant women as healthy as those in the Intergrowth 21st study [43].

## Conclusion

The agreement between Intergrowth 21st and Salomon equations, besides HC, was poor. This would imply different clinical decisions regarding the management of the pregnancy and the delivery. Encouraging women to attend antenatal clinics earlier and to use preventive measures against malaria such as long-lasting insecticidal bed nets, would probably be much more beneficial than just dating gestation or determining fetal size.

## Supplementary information

**Additional file 1: Supplemental Table 1.** Agreement and reliability of fetal biometrics z scores using Intergrowth 21st and Salomon equations.

## Data Availability

Data supporting the findings are not publicly available. Access could be granted upon motivated request to Tinto Halidou.

## Abbreviations

AC: Abdominal circumference
BPD: Biparietal diameter
FL: Femur length
HC: Head circumference
ICC: Intraclass correlation coefficient
INTERGROWTH – 21st: International fetal and newborn growth consortium for the twenty-first century
LLIN: Long lasting insecticidal net
LOA: Limits of agreement
PREGACT: Safe and efficacious artemisinin-based combination treatments for African pregnant women with malaria
