# Supplementary material for: Fetal biometry assessment with Intergrowth 21st’s and Salomon’s equations in rural Burkina Faso
Source: BMC Pregnancy Childbirth. 2020 Aug 26;20:492. doi: 10.1186/s12884-020-03183-5 (PMC7449020; doi:10.1186/s12884-020-03183-5)
Supplement: Supplementary file 1 — Additional file 1: Supplemental Table 1. Agreement and reliability of fetal biometrics z scores using Intergrowth 21st and Salomon equations. [file 12884_2020_3183_MOESM1_ESM.docx]

| **Supplemental Table 1**  Agreement and reliability of fetal biometrics z scores using Intergrowth 21^st^ and Salomon equations | | | | | |
| --- | --- | --- | --- | --- | --- |
|  | Mean ± SD | p-value^*^ | Median (IQR) | p-value^†^ | Range |
| Biparietal diameter |  |  |  |  |  |
| Intergrowth 21 | -0.62 ± 0.74 |  | -0.63 (-1.08 ; -0.15) |  | -3.40 ; 3.11 |
| Salomon | -0.27 ± 0.65 |  | -0.30 (-0.67 ; 0.16) |  | -2.68 ; 2.81 |
| Difference | -0.35 ± 0.18 | < 0.001 | -0.39 (-0.45 ; -0.31) | < 0.001 | -0.72 ; 0.33 |
| LOA | -0.70 ; 0.00 |  |  |  |  |
| ICC | 0.86 |  |  |  |  |
| Abdominal circumference |  |  |  |  |  |
| Intergrowth 21 | 0.98 ± 1.06 |  | 0.88 (0.31 ; 1.46) |  | -2.07 ; 9.90 |
| Salomon | 0.31 ± 0.57 |  | 0.28 (-0.02 ; 0.59) |  | -1.37 ; 5.22 |
| Difference | 0.67 ± 0.54 | < 0.001 | 0.57 (0.33 ; 0.90) | < 0.001 | -0.70 ; 4.68 |
| LOA | -0.40 ; 1.74 |  |  |  |  |
| ICC | 0.56^‡^ |  |  |  |  |

| **Supplemental Table 1 (continued):** Agreement and reliability of fetal biometrics z scores using Intergrowth 21^st^ and Salomon equations | | | | | |
| --- | --- | --- | --- | --- | --- |
|  | Mean ± SD | p-value^*^ | Median (IQR) | p-value^†^ | Range |
| Head circumference |  |  |  |  |  |
| Intergrowth 21 | 0.75 ± 0.78 |  | 0.72 (0.24 ; 1.27) |  | -1.56 ; 4.24 |
| Salomon | 0.76 ± 0.57 |  | 0.71 (0.40 ; 1.14) |  | -0.94 ; 3.36 |
| Difference | -0.01 ± 0.31 | 0.79 | -0.06 (-0.21 ; 0.16) | 0.11 | -0.62 ; 1.01 |
| LOA | -0.60 ; 0.59 |  |  |  |  |
| ICC | 0.90 |  |  |  |  |
| Femur length |  |  |  |  |  |
| Intergrowth 21 | 1.17 ± 1.00 |  | 1.27 (0.63 ; 1.77) |  | -2.44 ; 4.00 |
| Salomon | 0.27 ± 0.78 |  | 0.34 (-0.13 ; 0.75) |  | -2.54 ; 2.57 |
| Difference | 0.90 ± 0.24 | < 0.001 | 0.92 (0.77 ; 1.05) | < 0.001 | 0.10 ; 1.53 |
| LOA | 0.43 ; 1.38 |  |  |  |  |
| ICC | 0.64 |  |  |  |  |

| **Supplemental Table 1 (continued):** Agreement and reliability of fetal biometrics z scores using Intergrowth 21^st^ and Salomon equations |
| --- |
| ** Paired t test p value.*  † *Wilcoxon signed ranks test p value.*  ^‡^ *Intraclass correlation calculation for abdominal circumference z scores excluded one participant with extreme values (9.9 using Intergrowth equations and 5.22 using Salomon equations).*  *LOA: limits of agreement; ICC: intraclass correlation coefficient.* |
